# Overexpression of *Arachis hypogaea AREB1* Gene Enhances Drought Tolerance by Modulating ROS Scavenging and Maintaining Endogenous ABA Content

**DOI:** 10.3390/ijms140612827

**Published:** 2013-06-19

**Authors:** Xiao-Yun Li, Xu Liu, Yao Yao, Yi-Hao Li, Shuai Liu, Chao-Yong He, Jian-Mei Li, Ying-Ying Lin, Ling Li

**Affiliations:** 1 Guangdong Provincial Key Lab of Biotechnology for Plant Development, College of Life Science, South China Normal University, Guangzhou 510631, China; E-Mails: xiaoyun5893@163.com (X.-Y.L.); liuxu@scbg.ac.cn (X.L.); lilab@scnu.edu.cn (Y.Y.); liyihao400@163.com (Y.-H.L.); shuailiu89@163.com (S.L.); hechaoyong4321@163.com (C.-Y.H.); lijianmei563@163.com (J.-M.L.); linyingying1378@163.com (Y.-Y.L.); 2 Molecular Analysis and Genetic Improvement Center, South China Botanical Garden, Chinese Academy of Science, Guangzhou 510650, China

**Keywords:** *AhAREB1*, transcription factor, drought stress, *Arachis hypogaea*

## Abstract

*AhAREB1* (*Arachis hypogaea* Abscisic-acid Response Element Binding Protein 1) is a member of the basic domain leucine zipper (bZIP)-type transcription factor in peanut. Previously, we found that expression of *AhAREB1* was specifically induced by abscisic acid (ABA), dehydration and drought. To understand the drought defense mechanism regulated by AhAREB1, transgenic *Arabidopsis* overexpressing *AhAREB1* was conducted in wild-type (WT), and a complementation experiment was employed to ABA non-sensitivity mutant *abi5* (abscisic acid-insensitive 5). Constitutive expression of *AhAREB1* confers water stress tolerance and is highly sensitive to exogenous ABA. Microarray and further real-time PCR analysis revealed that drought stress, reactive oxygen species (ROS) scavenging, ABA synthesis/metabolism-related genes and others were regulated in transgenic *Arabidopsis* overexpressing *AhAREB1*. Accordingly, low level of ROS, but higher ABA content was detected in the transgenic *Arabidopsis* plants’ overexpression of *AhAREB1*. Taken together, it was concluded that AhAREB1 modulates ROS accumulation and endogenous ABA level to improve drought tolerance in transgenic *Arabidopsis*.

## 1. Introduction

Plant productivity is greatly affected by environmental stresses, including drought, low temperature and high salinity. Particularly, water stress is an important abiotic stress that leads to reduction of crop yields and affects agricultural productivity in many parts of the world. Peanuts, or groundnuts, are important oil and economic crops around the world and necessary for the national economy of China. In China, 70% of the peanut production areas are subject to varying degrees of water deficit that severely impact peanut production [1]. Therefore, studies on improvement of drought resistance of peanut are urgent and necessary. However, not much is known about drought stress responses, due to the complex genome (tetraploid) and the lack of efficient transgenic technology in peanut.

Numerous stress responsive genes that are involved in protecting plants far from stress-induced damages have been identified [2–4]. Abscisic acid (ABA), a universal stress response signal molecule, alters numerous transcriptome through dependent and independent pathways to enhance stress tolerance and it is involved in ion homeostasis and enhanced antioxidant defense [5,6]. Under stress conditions, ABA signaling is activated and phosphorylates downstream substrates, AREBs/ABFs, then stimulates ABA-responsive gene expression and ABA-related responses in plant [7]. AREB/ABFs are the member of basic domain leucine zipper (bZIP) transcription factors. In *Arabidopsis*, they include *AREB1/ABF2*, *AREB2/ABF4*, *ABF3*, *ABI3* and *ABI5*, *etc.*, which have been identified and characterized as ABA-responsive signaling molecular or downstream transcription factors [5,8–10]. Functional characterizations of AREB/ABFs have revealed that they are induced in response to drought, high salinity or even ABA treatment [11–13]. In *Arabidopsis*, AREB1/ABF2, AREB2/ABF4 and ABF3 do not completely overlap in their functions and can form homo- or hetero-dimers with each other [14]. Therefore, each AREB protein may play a specific role in response to water stress. Many downstream genes of AREB/ABFs have been illustrated, including *RD29B*, *AIL1*, *RAB1* and *RD20*, *etc.* [13,14]. In addition, AREB homologous genes, *ABP9* (isolated from *maize*) and *PtrABF* (isolated from *Poncirus tirfoliata*), have been reported to modulate cellular levels of ROS (reactive oxygen species) [15,16]. ROS has a dual role in various cellular compartments. It acts as a signaling molecule to process plant protective stress responses at low concentrations, and it causes oxidative damage at higher concentrations [15,17]. It has been well known that ABA regulates ROS producing and ROS scavenging genes to modulate the ROS levels [15]. AREB protein may play a crucial role in these proceedings.

AREB/ABF-related pathways and multiple mechanisms appear to contribute to increasing stress tolerance in *Arabidopsis* and other species, which is useful to understand the drought stress response in peanut. We have previously isolated a dehydration inducible AREB-like gene, *AhAREB1*, from peanut and found that *AhAREB1* gene was specifically induced by ABA, dehydration and drought [18]. Like others, *AREBs/ABFs* and *AhAREB1* genes encode a transcription activator [19], and the amino acid sequence of AhAREB1 shared homology with other AREBs/ABFs [18]. However, the function and exact mechanisms of *AhAREB1* in plant are still not clear.

Here, we report that the constitutively expressed *AhAREB1* gene is associated with high tolerance to dehydration and drought stresses in *Arabidopsis*, also recovering the ABA-sensitivity in *abi5* (ABA insensitive mutant). The potential AhAREB1 target genes were identified by comparing large-scale gene expression profiles of transgenic *Arabidopsis* under normal growth conditions under drought stress. According to the microarray results, expression patterns of candidate genes were further confirmed by quantitative PCR. Moreover, accumulation of ROS and ABA content was analyzed in *AhAREB1*-overexpressing plants.

## 2. Results

### 2.1. The AhAREB1 Overexpression Greatly Improves Drought Tolerance and ABA Sensitivity in Transgenic *Arabidopsis* Plants

**Figure 1:**
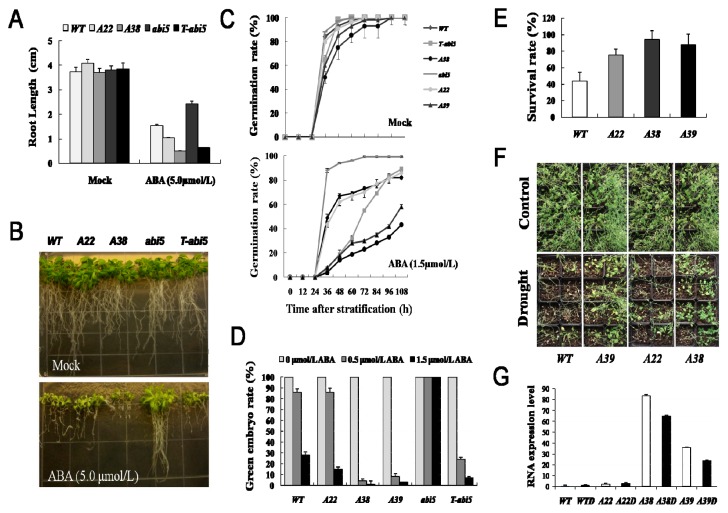
Drought tolerance and abscisic acid (ABA) sensitivity of *AhAREB1*-overexpressed plants. (**A**) Quantification of relative primary root growth length of seedlings treated with 5 μmol/L ABA at 14 day after stratification. Bars indicate standard deviation, *n* = 30; (**B**) Photographs of seedling at 20 day after transfer to control agar plates (Mock) or plates containing 5 μmol/L ABA; (**C**) Germination rate of seedlings on MS agar plate containing indicated concentration of ABA; (**D**) The green cotyledon of seedlings was analyzed after treatment with 0.5 or 1.5 μmol/L ABA; (**E**) The survival rate of wild-type (WT) and transgenic plants (*A22* and *A38* lines) were calculated after water deprivation; (**F**) Photographs showing plants after control and drought stress treatment. Watering was withheld from four-week-old plants for 10 d; thereafter, plants were rewatered for 4 d before the photograph was taken; (**G**) Real-time PCR detected the expression of *AhAREB1* genes in transgenic plants (*A22*, *A38* and *A39*) and WT plants at normal conditions and dehydration for 0.5 h, respectively. All experiments were performed in triplicate, and a representative result is shown. The error bars represent standard deviations (*n* = 20). WT represented wild-type plants; *A22* represented *A22* transgenic lines; *A38* represented *A38* transgenic lines; *A39* represented *A39* transgenic lines; *abi5* represented the abscisic acid-insensitivity mutant, *abi5-1; T-abi5* represented the plants that transformed *AhAREB1* into the *abi5* line. *WTD*, *A22D*, *A38D* and *A39D* represented the plants of *WT*, *A22*, *A38* and *A39* that were kept dehydrated for 0.5 h.

To examine the role of *AhAREB1* in plants, we performed *Arabidopsis* transformation using plasmid *p35S::AhAREB1* in which the gene was under the control of the cauliflower mosaic virus (CaMV), 35S promoter. Twenty four independent transgenic lines were identified in which the T2 plants segregated 3:1 for hygromycin resistance, and the hygromycin resistant T3 homozygous plants were then screened and confirmed by one-step RT-PCR analysis (data not shown). Three independent homozygous lines (*A22*, *A38* and *A39*) were chosen for investigation. Among these transgenic lines, *A22* displayed low-level expression of *AhAREB1*, while *A38* and *A39* showed high-level expression of *AhAREB1* (Figure 1G). To evaluate the ABA sensitivity, the non-sensitivity mutant, *abi5* (abscisic acid insensitive 5-1, *abi5-1*), was used as control, and the transgenic lines *T-abi5* (transformed *AhAREB1* into *abi5* line, named *T-abi5*) were used to determine recovery of ABA sensitivity. The growth of transgenic plants, expression of *AhAREB1*, germinating seeds, green cotyledon and axial root length were detected, respectively. There was no significant difference in growth phenotype between transgenic plants and wild-type (WT) at the seedlings stage. However, we observed a slight growth inhibition in *A38* and *A39* plants on the soil-grown after six weeks (Supplementary File 1, Figure S1 and Table S1). During the germination process, no significant difference was observed between *A22*, *T-abi5*, *abi5* and WT under normal growth conditions. However, *A38* and *A39* plants showed a low germination rate. Germination of *A22* and *A38* plants was more inhibited by ABA than WT (Figure 1C). In addition, after transforming the *AhAREB1* into *abi5*, all of them improved their ABA sensitivity under 1.5 μmol/L of ABA, which indicates that *AhAREB1* recovered the ABA sensitivity in *abi5* (Figure 1C). Similar results were observed in a green cotyledon and primary root growth experiment. Under normal growth conditions, the green cotyledon of all transgenic plants were 100%, but decreased dramatically after 1.5 μmol/L ABA treatment in *A38*, *A39* and *T-abi5* plants, while *abi5* plants showed non-sensitivity to ABA (Figure 1D). An ABA inhibition of primary root growth was observed for WT, *A22*, *A38*, *abi5* and *T-abi5* plants. *A38* and *T-abi5* plants were significantly inhibited by 5.0 μmol/L ABA, while *A22* and WT were less inhibited. On the other hand, *abi5* lines showed higher resistance to ABA than other lines (Figure 1A,B). The above findings demonstrated that overexpressed *AhAREB1* improves ABA sensitivity. Furthermore, the survival rate of *A22*, *A38* and *A39* plants was markedly higher in comparison with WT plants. After rehydration, the *A22*, *A38* and *A39* plants achieved 68%, 88% and 84% survival rates, respectively, while WT had only 40% (Figure 1E,F). The results also indicated that overexpressed *AhAREB1* elevated drought tolerance in the plants.

### 2.2. Identification of Target Genes of AhAREB1 Using Genome-Wide Microarray Expression Analysis

To identify the target genes of *AhAREB1*, we compared the expression profiles of 14 day-old *A38* plants with those of WT plants under both normal and stress conditions (dehydration 0.5 h) using the *Arabidopsis* gene expression 385k arrays (Roche NimbleGen). Genes 493 and 317 show differential expression between *A38* and WT plants under control growth conditions and dehydration stress (A38-CK *vs.* WT-CK or A38-dry *vs.* WT-dry), respectively. Genes 749 and 986 show difference in expression in *A38* plants and WT plants under the control condition compared with dehydration stress (A38-CK *vs.* A38-dry or WT-CK *vs.* WT-dry), respectively (Supplementary File 2).

The functional classification of differences in gene expression between *A38* and WT plants under control growth conditions were grouped into GOseq functional categories (http://www.geneontology.org). In *A38* plants, the differences expression genes are distributed under four main categories of cellular process (12.56%), physiological process (12.56%), catalytic activity (9.69%) and metabolism (8.59%) (Figure S2). Among these difference expression genes, *RD29A* (AT5G52310), *RD29B* (AT5G52300), *RD26* (AT4G27410) and *RD20* (AT2G33380) have been reported as stress responsive genes; *CAT2* (Catalase 2, AT4G35090), *CCS* (copper chaperone for SOD1, AT1G12520) and *CSD3* (copper/zinc superoxide dismutase 3, AT5G18100) involve increasing antioxidant capability; *NCED3* (nine-cis-epoxycarotenoid dioxygenase 3, AT3G14440), *ABA1* (zeaxanthin epoxidase, AT5G67030), *AtABCG40* (AT1G15520) and *CYP707A2* (abscisic acid 8′-hydroxylase, AT2G29090) participate in ABA biosynthesis, transporter or metabolism. Under normal conditions, except for *NCED3* and *AtABCG40*, all the above genes were significantly upregulated in *A38* plants in comparison to WT plants (A38-CK *vs.* WT-CK, ratio > 1.5-fold). After dehydration, *CSD3*, *ABA1*, *ABA3* and *AtABCG40* show no significant difference in expression, both in A38 plants and WT plants, while other genes, including *RD29A*, *RD29B*, *RD26*, *RD20*, *CAT2*, *NCED3* and *CYP707A2*, displayed significantly different expression in comparison to normal conditions (Supplementary File 1, Table S2). Furthermore, we analyzed transcriptional levels of the responsive genes to different hormones and environmental cues using the data sets available from Genevestigator (http://www.genevestigator.com/). The heat map is shown in Figure S3A. Comparing the Genevestigator stimulus data sets with our microarray data, we found that most of the genes showing increased expression levels in the *A38* lines are responsive to ABA, dehydration, osmotic and or salt stress in *Arabidopsis*. Meanwhile, most of these genes’ (such as *RD29A*, *RD29B*, *RD26*, *RD20*, *NCED3*, *CAT2* and *CYP707A2*) promoter region carries ABRE sequences: *ACTGT* (Supplementary File 1, Figure S3B).

**Figure 2:**
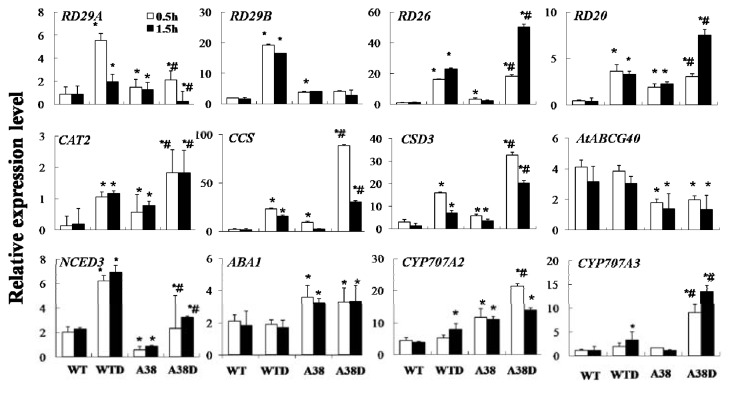
Expression analyses of related genes in *A38* lines and wild-type. The transcript level of *RD29A*, *RD29B*, *RD26*, *RD20*, *ABA1*, *AtABCG40*, *NCED3*, *CYP707A2*, *CYP707A3*, *CAT2*, *CCS* and *CSD3* were examined by quantitative PCR analysis in 14 day-old plants. *A38* and WT seedlings were grown on MS agar medium with or without dehydration treatment at the time points indicated. *18SrRNA* gene expression level was used as an internal control. Error bars represent standard deviation among the three reduplicate experiments. WT represents wild-type plants under normal conditions; WTD represented wild-type plants that were treated with dehydration for 0.5 h or 1.5 h; A38 represented *A38* lines under normal conditions; A38D represented *A38* lines that were treated with dehydration for 0.5 h or 1.5 h. * indicated that values of the WTD, A38 and A38D were significantly different from those of WT with *p <* 0.05, after dehydration for 0.5 h or 1.5 h, respectively. ^#^ indicated that values of A38D were significantly different from A38 at *p <* 0.05 after dehydration for 0.5 h or 1.5 h, respectively.

In addition, we confirmed the expression of these genes using quantitative PCR (q-PCR). Under dehydration, the expression pattern of *RD26*, *RD20*, *CAT2*, *NCED3*, *CCS* and *CSD3* is similar, which was strongly inducted at 0.5 h or 1.5 h both in *A38* plants and WT plants. The expression of *RD29A* was more strongly induced in WT plants than *A38* plants after dehydration for 0.5 h, which showed a dramatic decrease after dehydration for 1.5 h. On the other hand, the expression levels of *RD29B*, *ABA1* and *AtABCG40* were not affected after dehydration for 0.5 h compared with the normal condition in *A38* plants, although *RD29B* and *ABA1* were more highly expressed in *A38* than WT plants. Meanwhile, under the normal condition, *NCED3* and *AtABCG40* transcripts were slightly inhibited in *A38* plants when compared to WT plants. These findings are almost consistent with the microarray results. In addition, *RD29B* was markedly induced in WT plants after dehydration, irrespective of duration, 0.5 h or 1.5 h. However, no significant difference in expression of *RD29B* was found in *A38* plants after dehydration, although its expression was higher in *A38* plants than WT plants under normal conditions (Figure 2). This result is not completely consistent with microarray results.

### 2.3. Effect of Constitutive Expression of AhAREB1 on ROS Levels and ABA Content in *Arabidopsis thaliana*

**Figure 3:**
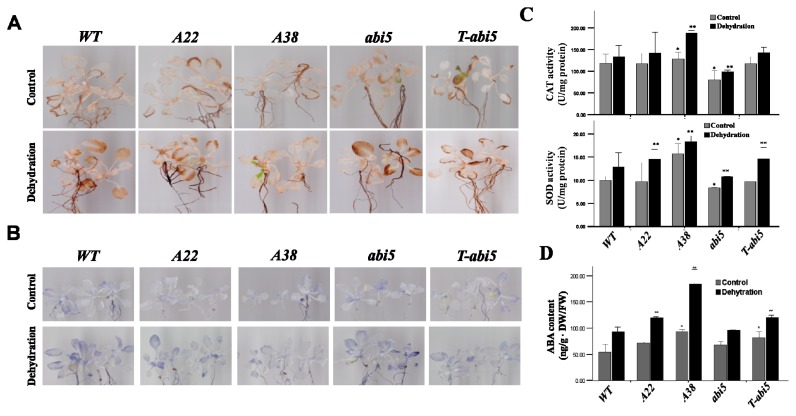
Constitutive expressions of AhAREB1 decreases ROS levels, but enhanced ABA content. (**A**,**B**) Twenty day-old plants were grown on agar plates, then dehydrated for 0.5 h. Cellular levels of H_2_O_2_ and O_2_^−^ were stained with 3,3-diaminobenzidin (DAB) and nitro blue tetrazolium (NBT) to visualize H_2_O_2_ and O_2_^−^, respectively; (**C**) Twenty day-old plants were grown on agar plates and then dehydrated for 0.5 h. Catalase (CAT) and superoxide dismutase (SOD) activities were calculated in WT, *A22*, *A38*, *abi5* and *T-abi5* plants. All experiments were repeated at least three times, and about 20 plants collected from seedlings were inspected in each experiment; (**D**) Endogenous ABA content was detected in whole plants of WT, *A22*, *A38*, *abi5* and *T-abi5*. Fourteen day-old plants were grown on soil for 14 days, then dehydrated for 10 days and were subsequently collected to measure endogenous ABA. Data are presented as the mean ± standard deviation. WT represented wild-type plants; *A22* represented *A22* transgenic lines; *A38* represented *A38* lines; *abi5* represented the abscisic acid-insensitivity mutant, abi5-1; *T-abi5* represented the plants that transformed *AhAREB1* into *abi5* lines.

To acquire further insights into *AhAREB1* regulation mechanism in plants under water stress, ROS levels and ABA levels were examined, associated with the related target genes of *AhAREB1*, from microarray expression analysis. Two prominent ROS species, O_2_^−^ and H_2_O_2_, were examined by nitro blue tetrazolium (NBT) and 3,3-diaminobenzidin (DAB) staining under normal growth and dehydration stress conditions, respectively. As shown in Figure 3A,B, the steady state levels of O_2_^−^ (visualized as dark blue products) and H_2_O_2_ (visualized as deep brown products) were reduced in the leaves of *A22*, *A38* or *T-abi5* plants compared to WT or *abi5* plants under normal conditions and dehydration. We also measured the activity of ROS-scavenging enzymes, catalases (CATs) and superoxide dismutases (SODs), in all plants under control conditions and dehydration. The CATs activities in *A38* plants were higher than that WT plants, not only under control, but also under dehydration conditions, while the CATs activities in *abi5* plants were lower than that WT plants (Figure 3C). Meanwhile, after dehydration, water stress caused significant increase of SOD activity in *A22*, *A38* and *T-abi5* plants (Figure 3C). These results demonstrate that *AhAREB1* plays a pivotal role in plant tolerance to drought stress by the controlling of ROS accumulation.

Based on microarray gene expression analysis, we found that many genes involved in ABA synthesis or metabolism were significantly differently expressed in *A38* plants. Therefore, we hypothesize that there would more ABA content and drought stress resistance in the *A38* plants. To examine this, ABA levels were measured in transgenic plants. The results show that *A38* and *T-abi5* have higher ABA content than WT or *abi5* plants under both conditions, normal or dehydrated (Figure 3D). These data suggest that the *AhAREB1* gene may improve endogenous ABA synthesis in transgenic plant, and the roles of target genes are consistent with stress resistant phenotypes of the transgenic plants.

## 3. Discussion

In our previous work, we have shown that *AhAREB1* gene, which shares high sequence homology with *GmAREB1*, *SlAREB* and *ABF2/AREB1*, responds to environmental stimuli [18]. Further study showed that AhAREB1 functions as a transcriptional activator in yeast [19]. Here, we showed that (1) *AhAREB1*-overexpressed plants had a higher survival rate under drought stress and were more sensitive to ABA than WT plants; (2) the ABA sensitivity in the *abi5* mutant could be recovered by *AhAREB1*; (3) ABA- and drought-responsive genes (such as *RD20*, *RD26*, *RD29A*, *RD29B*), ABA homeostasis genes (*ABA1*, *AtABCG40*, *NCED3* and *CYP707A2*) and ROS scavenging genes (*CAT2*, *CCS*, *SOD1* and *CSD3*) were differentially expressed between *AhAREB1*-overexpressed plants and WT plants; (4) constitutive expression of *AhAREB1* reduces ROS levels by increasing the CATs and SODs activity, but improving the ABA content in *Arabidopsis*.

Previous findings showed that AREB/ABF transcription factors are positive regulators of ABA signaling in response to drought stress [8,12]. Also, overexpression of the *AREB*/*ABF* genes or stress-related genes in plants can result in slight growth inhibition [10,20–22]. Our results shown in Supplementary File 1, Figure S1A and Table S1 are consistent with these previous reports. The phytohormone, ABA, not only regulates abiotic stress response, but also imparts growth retardation [5], because it can reprogram the transcriptional events widely in plants via AREB/ABF transcription factors [8,12]. As expected, many ABA- and drought-responsive genes were upregulated in the *AhAREB1*-overexpressed plants. They included *RD20*, *RD26*, *RD29A* and *RD29B* genes, and so on, which are known to function in ABA-mediated stress-signal transduction [23,24]. Besides, higher survival rate and ABA sensitivity were observed in the *AhAREB1*-overexpressed plants, suggesting that plants have higher tolerance or resistance to stress [25]. Further, transformation of *AhAREB1* into *abi5* recovered the ABA sensitivity of the mutant plants. The protein, ABI5, also is a bZIP transcription factor, which acts as a central regulator of ABA signaling in *Arabidopsis thaliana* [26,27]. Previous research has shown that *AREB/ABF* and *ABI5* are homologous protein families [28]. Our results suggest that AhAREB1 functions in ABA signal transduction and may overlap with ABI5 roles redundantly. In this study, we did not detect any difference in expression of *ABI5* in *A38* plants, using microarray under control conditions or dehydration stress (Supplementary File 2), while the expression level of *ABI5* increased in WT under dehydration stress.

It is well known that ROSs act as intracellular messengers [29–32]. Conversely, ROS, when accumulated in excess, causes damage to plants [15,31]. Therefore, appropriate regulation of ROS levels is important to improve tolerance to abiotic stress. Previous studies indicated that ABA via ABRE transcription factors regulates ROS-producing and ROS-scavenging genes, such as *SODs*, *APXs* and *CATs* genes, to modulate the cellular ROS levels [32–35]. It has been known that constitutive expression of maize *ABRE2* orthologous gene, *ABP9*, causes reduced cellular levels of ROS and enhances drought stress tolerance [15]. In this study, constitutive expression of *AhAREB1* in *Arabidopsis* enhanced the ROS scavenging genes (*CAT2*, *CCS*, *SOD1* and *CSD3)* expression, also reducing cellular ROS levels and maintaining higher CAT and SOD activity than the WT plants at normal and dehydration conditions. These results clearly indicate that the *AhAREB1* gene is involved in modulation of ROS accumulation in *Arabidopsis* and enhances tolerance to dehydration, which is consistent with the previous reports [15].

Several genes encoding enzymes for ABA biosynthesis or catabolic activities were altered in *AhAREB1*-overexpressed plants. NCED3 is a key enzyme of ABA biosynthesis under drought stress [36] in *Arabidopsis*, whose transcription level shows rapid response to dehydration or drought stress and gradually decreases following the time of stress [37,38]. ABA1 is an enzyme important in *de novo* ABA biosynthesis, which exhibited drought stress resistance, while overexpressed in *Arabidopsis* plants [39]. In *A38* transgenic plants, *NCED3* was downregulated in expression under control conditions, but upregulated under dehydration, both in WT and *A38* plants. However, *ABA1* was upregulated under control conditions and had no significant difference in expression under dehydration, either in *WT* or *A38* plants. It also indicated that *AhAREB1* might participate in the negative regulation of *NCED3* gene transcription. Several ABRE-like elements are found in the *NCED3* promoter, given that AhAREB1 protein may be capable of activating *NCED3* via specific interactions with ABREs. Meanwhile, CYP707A2 belongs to ABA 8′-hydroxylase and has a significant role in ABA catabolism during imbibitions and regulates seeds dormancy [40–42]. In *Arabidopsis*, four members (CYP707A1-A4) were identified as the enzymes responsible for catalyzing ABA 8′-hydroxylation [40–43]. From their tissue specificity or expression patterns, CYP707As may have distinctive roles in plants. For example, *CYP707A3* was involved in dehydration and rehydration response [44]. Our microarray data also showed that *CYP707A3* was greatly induced by dehydration both in WT (>3-fold) and *A38* (>2-fold) plants (Supplementary File 2). Here, *CYP707A2* seemingly responded only to ABA content or *AhAREB1*, for its transcripts always were induced in *A38* plants whenever under control conditions or drought stress conditions. ABA content is associated with drought stress resistance in plants during the early stage [5,32,45,46]. These genes are thought to play a major role in maintaining a balance of endogenous ABA content. Therefore, detection of ABA content is very important and necessary in *A38* plants. It is noteworthy that ABA content was higher in *A38* transgenic plants than WT, both under control conditions and dehydration. Combined with the gene expression data, it suggests that endogenous ABA was mainly synthesized by ABA1 and NCED3, under control condition and drought stress, respectively, in our transgenic plants. Although *CYP707A2* expression was upregulated in *A38* plants, both under control and stress conditions, it may be a feedback loop between its transcription and ABA content/ABA signaling by AhAREB1 modulation. In fact, *CYP707A2* plays a central role during seed imbibition and regulates dormancy [42,43], and its transcription was not induced by dehydration or rehydration [44]. It simply means that the high expression of *CYP707A2* was not associated with stress resistance or only has a small role in ABA catabolism during the seedling stage. Under dehydration, the endogenous ABA content was further regulated by a balance between biosynthetic and catabolic activities through NCED3 and CYP707A3. The *AtABCG40* gene encodes a plasma membrane ABA uptake transporter and is necessary for timely responses to ABA [47]. In *A38* plants, the transcription of *AtABCG40* was downregulated at the whole plant level. This suggested that ABA transporter rate may be lower in *A38* plants than in WT plants. As a result, *A38* plants keep a high ABA content and show stress resistance.

## 4. Experimental Section

### 4.1. Plant Materials

Seeds of peanut (*Arachis hypogaea* L. cv. Shanyou 523) were sown in growth medium (vermiculite, perlite and soil; 1:1:2) and grown in plastic pots in a growth chamber with a photoperiod of 16 h light at 26 °C and 8 h of darkness at 22 °C, as described previously [48]. Seeds of *Arabidopsis* wild-type (WT) were surface sterilized in 70% ethanol for 2 min and in 1% sodium hypochlorite for 10 min. After five washes with sterile water, the seeds were sown on MS (Murashige and Skoog) medium supplemented with 2% sucrose and 0.8% agar. After 2 days of vernalization at 4 °C, the seeds were germinated and grown in a growth chamber under a daily cycle of 16 h light and 8 h dark at 20 ± 2 °C. Seven days after sowing, the seedlings were planted in plastic pots in a medium of vermiculite, peat moss and perlite (1:2:1).

### 4.2. Plasmid Construction and *Arabidopsis* Transformation

To generate the overexpression construct of the *AhAREB1* coding region, full-length cDNA was generated by RT-PCR with the following primers: 5′-CTG AGATCT ATG AAC TTC AGG GGC TAT GGT GAT-3′ and 5′-CTGGGTGACC CTA CCA GGG ACC TGT AAC TGT CCTT-3′ (the underlined parts are BglII and BstEII sites, respectively). The PCR product, confirmed by DNA sequencing, was cloned into modified binary vector pCAMBIA1301 between the BglII and BstEII sites, under the control of the constitutive cauliflower mosaic virus 35S promoter to generate the *35S::AhAREB1* frame. The overexpression construct was introduced into *Agrobacterium tumefaciens* strain GV3101 and then transformed into *Arabidopsis* WT plants by the floral dip method. Transgenic T1 lines were selected on MS medium containing 40 mg/L kanamycin (Sigma-Aldrich, St. Louis, MO, USA). T2 seeds from the selected transgenic plants were germinated on a medium with 40 mg/L hygromycin, and then the homozygous lines were selected. The homozygous T3 progeny were then examined for the expression level of the target genes by One Step SYBR PremeScript RT-PCR kit (Takara Biotechnology, Dalian, China) using two specific primers, smRT-AhAREB1-F and -R (Supplementary File 1, Table S3).

### 4.3. Assays of Seed Germination and Green Cotyledons; Growth of Roots

Seed germination, green cotyledons and root growth were treated with exogenous ABA and assayed, as described previously [48]. Sterilized seeds of transgenic *Arabidopsis* and WT were sown on 1/2 MS agar plates, which were supplemented with 1.5 μmol/L ABA to observe germination. To score green cotyledons, the agar plates were supplemented with 0.5 and 1.5 μmol/L ABA, and germinated seeds with fully expanded green cotyledons were recorded on day 4 after sowing. Three independent experiments were performed (30 seeds in each line per test). Assays of root growth were carried out by transferring 4 days-old seedlings of transgenic *Arabidopsis* and the WT, under normal sprouting, onto 1/2 MS medium agar plates with 5 μmol/L ABA treatments. Primary root lengths of more than 20 seedlings in each line were measured on day 8 after the start of treatment. The experiment was triplicated.

### 4.4. Drought Stress Tolerance Assays

Survivability tests under the drought conditions were conducted on three independent transgenic lines and the wild-type. Seeds were surface-sterilized and cultivated on 1/2 MS medium for 10 days after radical emergence and then were transferred to soil. At the rosette stage (4 weeks after sowing), half of the samples were subjected to drought stress. These samples were subjected to drought stress by withholding irrigation for 10 days to evaluate drought tolerance visually (the leaves withered). These samples were then rewatered (once a day) for 4 days. The remaining half of the samples was grown under a standard irrigation regime (watering once a day) for 14 days, as a control. The survival rates of the plants were calculated as the ratio of plants with green leaves to the total number of plants used. All experiments were repeated at least 3 times, and more than 32 plants of each line were used in each replicate.

### 4.5. Microarray Analysis

Two-week-old *A38* lines and WT grown on the plate were harvested directly or after dehydration treatment for 0.5 h by opening the lids of the plates directly, and they were subjected to microarray experiments using the *Arabidopsis* Gene expression 385k Arrays (Roche NimbleGen, Madison, WI, USA). Total RNA isolated using TRIzol reagent (Takara, Dalian, China) was used for the preparation of Cy5-labeled and Cy3-labeled cDNA probes. All microarray experiments, including data analysis, were carried out as described previously [49]. The reproducibility of microarray analysis was assessed by dye swap in each experiment.

### 4.6. Quantitative PCR Assay

Total RNA was isolated by TRIzol reagent (Invitrogen, Carlsbad, CA, USA) from each plant sample. About 2 μg of DNaseI-treated and purified total RNA was reverse transcribed with SUPERSCRIPTTM III Reverse Transcriptase (Invitrogen, Carlsbad, CA, USA) in a reaction volume of 20 μL to generate the first-strand cDNA, according to the manufacturer’s instructions. Real-time PCR was performed using an Optical 96-well Fast Thermal Cycling Plate with an ABI 7500 real-time PCR system. Each reaction contained 10 μL of 2× SYBR Premix Ex TaqTM (Takara, Dalian, China), 20 ng of cDNA and 0.1 μmol/L of gene specific primers in a final volume of 20 μL. The thermal cycle used was: 95 °C for 30 s, then 40 cycles at 95 °C for 5 s and 60 °C for 34 s. The target genes were *AhAREB1*, *ABA1*, *AtABCG40*, *NCED3*, *CYP707A2*, *CYP707A3*, *CAT2*, *CCS*, *CSD3*, *RD26*, *RD20*, *RD29A* and *RD29B*. An internal control gene of *18SrRNA* was used for quantitative PCR with gene specific primers (Supplementary File 1, Table S3). The relative expression levels were calculated using the relative 2^−ΔΔCT^ method and determined as described previously [48].

### 4.7. *In Situ* NBT Staining and Measurement of SOD Activity

*In situ* accumulation of superoxide (O_2_^−^) was examined based on histochemical staining by nitro blue tetrazolium (NBT). For O_2_^−^ detection, the treated samples were immersed in 1 mg/mL fresh NBT solution (prepared in 10 mmol/L phosphate buffer, pH 7.8) and incubated in light at 25 °C until dark spots were observed. The stained samples were then bleached in concentrated ethanol and kept in 70% ethanol.

For extraction of superoxide dismutase (SOD, EC 1.15.1.1), about 0.5 g of leaf sample was ground in liquid nitrogen with a pre-cooled pestle and mortar and homogenized in 5 mL of extraction buffer containing 50 mmol/L phosphate buffer (pH 7.8) and 1% polyvinylpyrrolidone (PVP). The homogenate was centrifuged at 10,000× *g* for 20 min at 4 °C, and the resulting supernatant was collected for enzyme activity analysis. Activities of SOD were spectrophotometrically measured using an SOD Detection Kit (A001, Jiancheng, Nanjing, China), according to the manufacturer’s instruction. Protein content was estimated using BCA Protein Assay Kit (P0012BCA, Beyotime, Shanghai, China), according to the manufacturer’s instruction. One unit of SOD activity is defined as the amount of protein. The results were expressed as enzyme activity per milligram protein (U/mg).

### 4.8. *In Situ* DAB Staining and Measurement of CAT Activity

*In situ* accumulation of hydrogen peroxide (H_2_O_2_) was examined based on histochemical staining by 3,3-diaminobenzidin (DAB). For H_2_O_2_ detection, the treated samples were immersed in 1 mg/mL fresh DAB solution (pH 5.8) (prepared in 10 mmol/L phosphate buffer, pH 3.8) and incubated in dark at 28 °C for 8 h. The stained samples were then bleached in 80% ethanol and boiled for several minutes (2–5 min), then dropped for staining by concentrated ethanol and kept in 4 °C, as described by Cheng and Song [50].

For extraction of catalase (H_2_O_2_ oxidoreductase; EC 1.11.1.6), about 0.5 g of leaf sample was ground in liquid nitrogen with 2–3 mL pre-cooled phosphate buffer (pH 7.0), then poured into a 25 mL volumetric flask with constant volume to scale. The mix slurry was kept at 5 °C for 10 min. The homogenate was centrifuged at 10,000× *g* for 15 min at 4 °C, and the resulting supernatant was collected for enzyme activity analysis. Activities of CAT were measured spectrophotometrically by UV absorption at 240 nm. Protein content was estimated using a BCA Protein Assay Kit (P0012BCA, Beyotime, Shanghai, China), according to the manufacturer’s instruction. One unit of CAT activity is defined as the amount of protein. The results were expressed as enzyme activity per milligram protein (U/mg).

### 4.9. Quantification of ABA Levels

To determine the ABA levels in WT and transgenic plants, 28 day-old seedlings were dehydrated for 10 days. ABA extraction and detection were performed, as previously described [51]. In brief, 300–500 mg of plant material was frozen in liquid nitrogen and ground using a Mixer-Mill. Plant material was treated with 1–2 mL of ABA extraction buffer (10 mmol/L HCl and 1% (*w*/*v*) polyvinyl polypyrrolidone in methanol). The mixture was kept at 4 °C overnight. After centrifugation, the supernatant was neutralized with 1 mol/L NaOH and ABA levels were quantified using the liquid chromatogram. Raw values for ABA levels were standardized by plant mass and extraction volume.

### 4.10. Subcellular Localization

For the localization of AhAREB1, the coding frame of *AhAREB1* prepared by PCR was cloned into the frame in front of the GFP coding region of modified pBI121 (Clontech, Mountain View, CA, USA) using the Xba1 and Sma1 sites. The construct was used to transform *Arabidopsis* (Col-0), and T1 plants were used to determine GFP localization. Roots of 10 day-old seedlings were examined for the green (GFP expression) fluorescence, employing a confocal microscope (LSM 5 Pascal, Zeiss, Jenaer, Germany).

## 5. Conclusions

In summary, we demonstrated that *AhAREB1* acts as a transcriptional activator of stress-relative, ROS-modulated genes and ABA-induced genes under drought or dehydration stress, and it may play an important role in drought stress tolerance via ABA homeostasis and control of ROS accumulation.

## Supplementary File 1
