# Supplementary material for: Overexpression of Arachis hypogaea AREB1 Gene Enhances Drought Tolerance by Modulating ROS Scavenging and Maintaining Endogenous ABA Content
Source: Int J Mol Sci. 2013 Jun 19;14(6):12827–42. doi: 10.3390/ijms140612827 (PMC3709814; doi:10.3390/ijms140612827)
Supplement: Supplementary file 1 [file ijms-14-12827-s001.pdf]

Supplementary File 1

Figure S1. Growth phenotype of plants. (**A**) Eight-week-old of Wild-type (*WT*), *abi5* (abscisic acid-insensitive mutant 5, *abi5*), *AhAREB1*-overexpressed plants (*A22*, *A38*, *A39*) and *AhAREB1*-transformed in *abi5* mutant plants (*T-abi5*) grown under normal condition; (**B**) Two-week-old seedlings of *WT*, *A22, A38*, *A39*, *abi5* and *T-abi5* plants grown on MS plate under normal condition. There was no significantly difference growth phenotype between transgenic plants and WT at seedlings stage, but slight growth inhibition was observed in *A38* and *A39* plants on the soil-grown after 6 weeks.

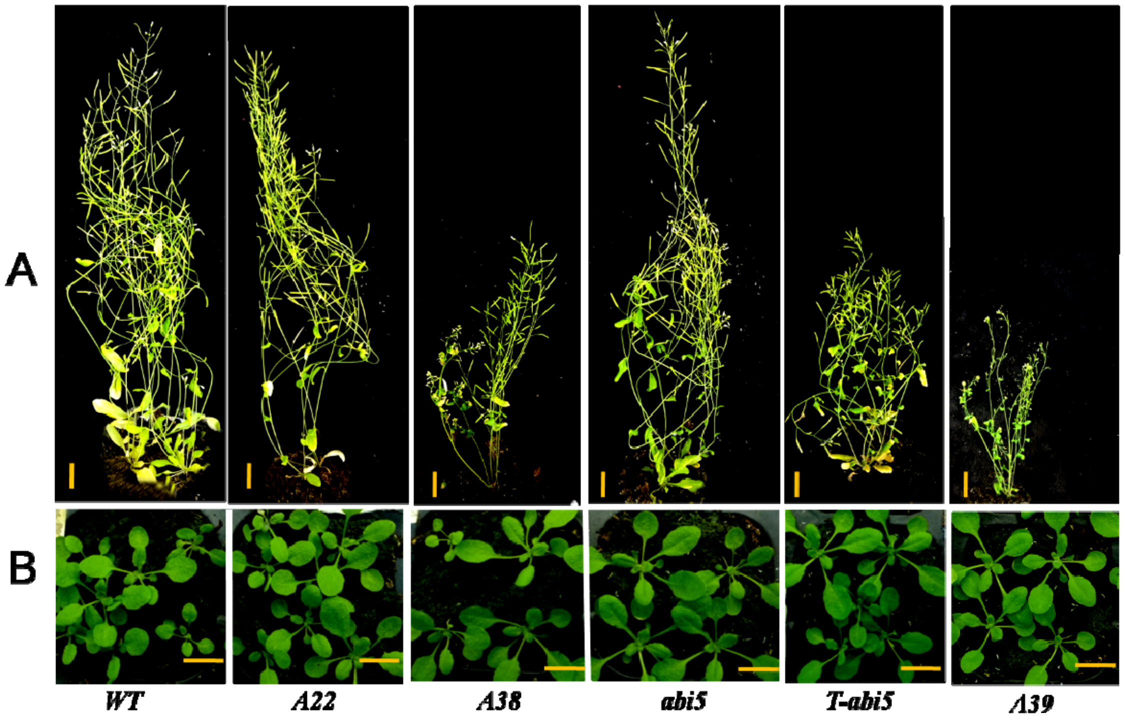

Table S1. Number, length and width of laminas in WT and transgenic *Arabidopsis.*

Line	No. of rosulate leaf	Average leaf length (cm)	Average leaf width (cm)	Plant height (cm)
WT	10.3 ± 0.6	1.1 ± 0.41	0.6 ± 0.31	27.6 ± 2.0
A22	11.1 ± 0.7	1.1 ± 0.28	0.7 ± 0.24	26.7 ± 1.1
A38	8.9 ± 1.0	0.8 ± 0.23 *	0.5 ± 0.42 *	21.5 ± 0.5 *
A39	9 ± 0.6	0.9 ± 0.14 *	0.6 ± 0.32 *	23.5 ± 0.2 *
Abi5	10.0 ± 0.8	1.0 ± 0.21	0.6 ± 0.22	25.5 ± 0.5
T-abi5	9.9 ± 0.9	0.88 ± 0.33 *	0.65 ± 0.12 *	24.5 ± 0.45 *

An asterisk (*) is used especially to indicate the significant difference ($p < 0.05$). All experiments were performed in triplicate and 20 plants at least were chosen at random to use for every lines.

Figure S2. Go annotation results of microarray expression analysis.

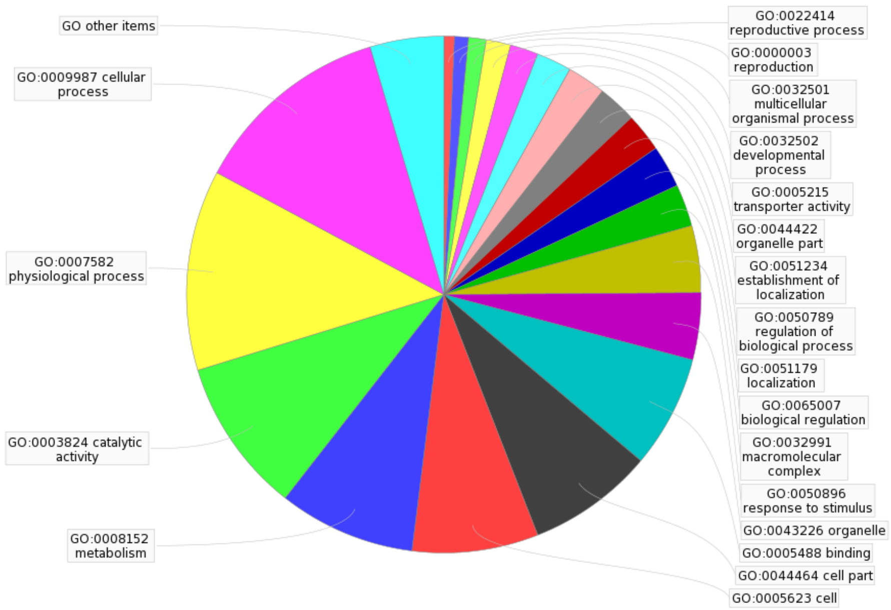

Figure S3. Regulation of differential expression of related genes to environmental cues. (**A**) The meta-profile analysis of the changes in transcript abundance of the 9 related genes in response to different stresses or environmental cues was established with the Genevestigator database (off note, *AtHSD2* was not found in genevestigator database). The gene expression responses are calculated as log2-ratios between signal intensities from different stress or environmental treatments compared to control or mock-treated samples. The resulting heatmap is color coded as indicated and reflects up-regulated (red color) or down-regulated (green color) genes. A grey color indicates that gene expression was not detectable in both treatment and control conditions; (**B**) Core motif of ABRE element in the promoters of these genes, shole promoter sequence were searched in both the forward and reverse strands of the promoter regions and distributed evenly. The red triangle indicated the direction of ABRE element.

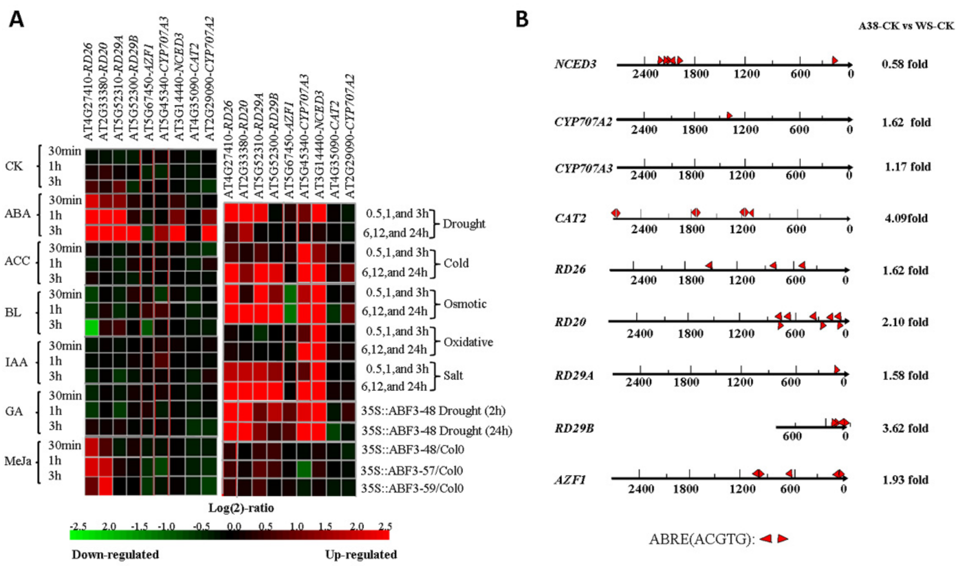

Figure S4. Subcellular localization of AhAREB1 protein in transgenic *Arabidopsis*. AhAREB1-GFP fusion proteins (**A**) or GFP control proteins (**B**) were expressed in *35S:AhAREB1-GFP* or *35S:GFP* transgenic Arabidopsis. Root from two-week-old seedlings was observed suing a confocal laser scanning microscopy (LSM 510 MET, Zeiss, Zeiss, Jenaer, Germany). (**A**,**B**) Fluorescent field of GFP; (**C**,**D**) Bright field.

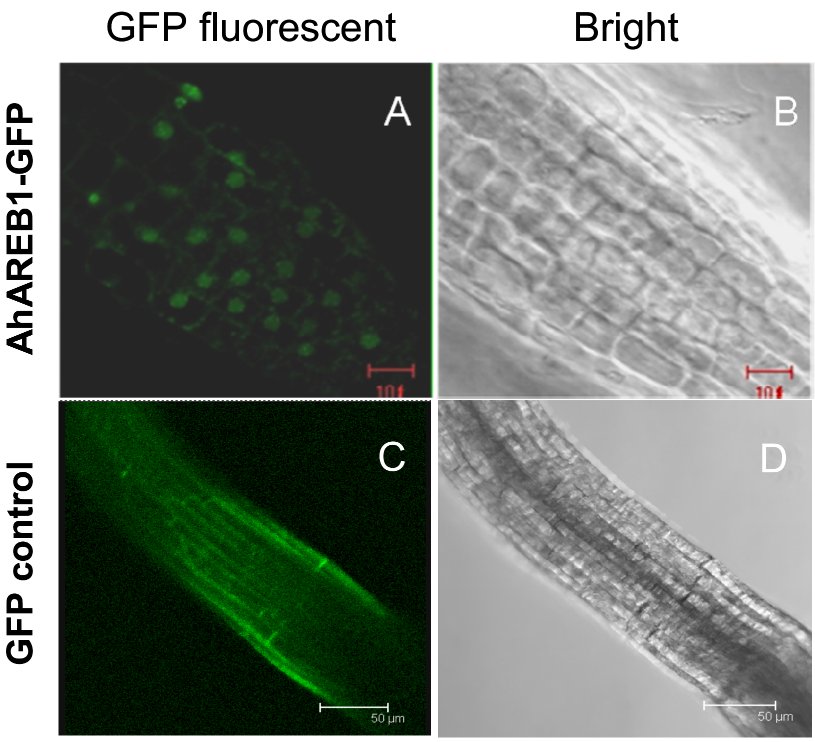

Table S2. Difference expression genes.

Gene ID	Gene symbol	A38-CK *vs.* WT-CK	A38_Dry *vs.* A38_CK	WT_Dry *vs.* WT_CK	A38_Dry *vs.* WT_Dry
AT4G35090	CAT2	7.53 *	2.51	4.09	5.95
AT5G18100	CSD3	1.96	-	-	-
AT1G12520	ATCCS	1.68	0.47	-	-
AT5G67030	ABA1	1.56	-	-	-
AT1G16540	ABA3	2.18	-	-	-
AT3G14440	NCED3	0.48	10.88	4.96	0.50
AT1G15520	AtABCG40	0.49	-	-	-
AT2G29090	CYP707A2	1.72	2.30	2.85	1.54
AT5G52310	RD29A	1.58	6.29	19.17	0.59
AT5G52300	RD29B	3.62	7.60	20.47	-
AT4G27410	RD26	1.94	24.70	16.39	-
AT2G33380	RD20	2.10	5.63	12.98	-

* fold change. - no significant difference.

Table S3. Primers for this study.

Primer name	Primer sequence (5' to 3')
smRT-AhAREB1-F	ATGAACTTCAGGGGCTATGGTGAT
smRT-AhAREB1-R	CTACCAGGGACCTGTAACTGTCCTT
RD26-F	TTACGGTGGTTACGATGCG
RD26-R	GAAACACCAAACCCACTCG
NCED3-F	CGGACGGAACTAAATCACC
NCED3-R	TCCCGAATCTTGCGACCTT
real-AhAREB1-F	ACAAGGGCAACCAGCATTAGG
real-AhAREB1-R	TCACCACCACCATACCAACCA
RD20-F	AGGAAGGTATGTCCCAGTT
RD20-R	CGATTTCCCTCGGTTACAT
CYP707A2-F	CACCACCGCTTCTGTCTTAAC
CYP707A2-R	TGCTGCTCTTAGTGTCTCTTGT
ATHSD2-F	GGCAAGCGTCTATTCAGCAA
ATHSD2-R	TGAGGAGTGGTCATATCCGTAG
CAT2-F	GTGTCTTCTCCTATGCCGATAC
CAT2-R	ATGGTGGTTGTTGTGGTGAG
CYP707A3-F	TGTCCAGGCAATGAGTTAGC
CYP707A3-R	CCAAGGCAATAGGCAATCCA
RD29A-F	ATCACTTGGCTCCACTGTTGTTC
RD29A-R	ACAAAACACACATAAACATCCAAAGT
RD29B-F	AAAACGTTTGGAGGAAGATCC
RD29B-F	GGTACTCCAGCTTCTCCACCT
18SrRNA-F	TACGTCCCTGCCCTTTGTAC
18SrRNA-R	CCTACGGAAACCTTGTTACGAC

© 2013 by the authors; licensee MDPI, Basel, Switzerland. This article is an open access article distributed under the terms and conditions of the Creative Commons Attribution license (http://creativecommons.org/licenses/by/3.0/).
